# Unveiling the potential: Harnessing spectral technologies for enhanced protein and gluten content prediction in wheat grains and flour

**DOI:** 10.1016/j.crfs.2025.101054

**Published:** 2025-04-12

**Authors:** Gözde Özdoğan, Aoife Gowen

**Affiliations:** School of Biosystems and Food Engineering, University College Dublin (UCD), Belfield, Dublin, D04 V1W8, Ireland

**Keywords:** Spectral imaging, Spectroscopy, Wheat, Protein, Gluten

## Abstract

Protein and gluten content is one of the most crucial quality characteristics in the wheat industry. However, these properties are measured after grinding wheat kernels into the flour. In this study, grain samples from 38 different wheat cultivars were collected, and their protein, wet and dry gluten content were measured traditionally. Spectral information was obtained using three non-destructive instruments, including benchtop visible-near infrared hyperspectral imaging (HSI), portable short wavelength infrared HSI and Fourier-Transform near-infrared spectroscopy from both whole grains and their flour samples. Partial least squares regression (PLSR) and Gaussian process regression (GPR) with three spectral pre-treatments were used to compare performances and Neighborhood Component Analysis was applied for wavelength selection.

Through HSI, wheat kernels revealed their protein and gluten content with remarkable precision, achieving R^2^_P_ values exceeding 0.97 using GPR based on whole kernel data utilising four wavelengths in the Visible range. The key novelty of this work is that it demonstrates the suitability of visible range hyperspectral imaging for direct prediction of protein and gluten with high accuracy, without the need for sample grinding, thus underscoring the significance of visible spectral information in determining protein and gluten-related parameters.

## Highlights

• Models based on whole kernels perform better than models based on flour data.
• Gaussian Process Regression performs better than Partial Least Squares Regression.
• Visible region is as efficient as broader regions such as NIR and SWIR.
• WG, DG and protein can be predicted by four wavelengths within the Vis range.

## Graphical abstract

**Figure:**
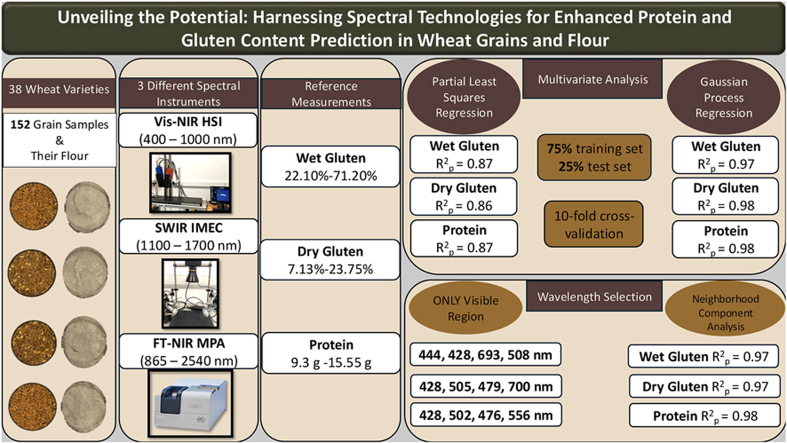

## 1 Introduction

The importance of wheat as a global food commodity is well recognised, with wheat being among the highest-produced grains, exceeding production volumes of 700 million metric tonnes (Shahbandeh, 2023). While about 90 % of the wheat grain produced is used for human consumption in different forms, such as bread, pasta, cake, and cookies, thanks to its nutritional value and functional diversity, the remainder has been used for seed, feed, and the production of industrial products including gluten, starch, and enzymes (Igrejas and Branlard, 2020).

One of the main features for determining wheat quality, utilisation, and marketing price is its protein content and composition. For instance, high-protein wheat kernels are usually used in the bread industry, whereas low-protein ones are in cookie production (Kanit et al., 2015). Wheat kernels can contain up to 15 % protein, and wheat proteins can be divided into storage and functional proteins. Storage proteins (gluten proteins) comprise 80–85 % of total proteins and are classified as gliadins and glutenins. In detail, gliadins can be categorised as α-gliadins, β-gliadins, γ-gliadins, and ω-gliadins while glutenins are subdivided into high molecular weight glutenin and low molecular weight glutenin (Biesiekierski, 2017). When wheat flour is kneaded by mixing with water, these gluten proteins combine by chemical bonds and form a rubbery matrix called gluten that can exhibit elastic and plastic properties. Gluten provides the main structure for the dough to rise by retaining carbon dioxide and gaining a porous texture (Dizlek, 2011). Gluten proteins show variability in components and size, depending on grain origin, climatic conditions, and production flow; this confers unique properties to the products it is used in. For example, different wheat varieties can create discrete gluten structures in the dough (Wieser, 2007).

The rheological and textural properties of dough, including elasticity, hardness, and porosity are highly correlated to the gluten content and strength. Therefore, the determination of gluten amount and composition of protein types is significant for the processed bakery product industry. Traditionally, gluten amount has been analysed by the “hand washing” method or using the glutomatic system which is a special equipment produced for this purpose (Wang et al., 2004). Analytical techniques such as gel electrophoresis, enzyme-linked immunosorbent assay (ELISA), and high-performance liquid chromatography (HPLC) have performed well in investigating gluten content in recent years. However, these methods are destructive, time-consuming, and require experienced analysts who can handle complicated sample preparation steps and comment on results objectively. But more importantly, they can be implemented only when the wheat is in the flour form after grinding (Haraszi et al., 2020). Because of these drawbacks, a particular need has emerged to evaluate the quality of wheat kernels in a rapid, environmentally friendly, and robust way. To fulfil this demand, studies related to spectroscopy, including nuclear magnetic resonance (NMR), fluorescence spectroscopy (FS) and more popularly, infrared spectroscopy (NIRS) have been researched in recent years (Delwiche, 2003; Dowell et al., 1999; Qiu et al., 2022) and achieved promising results. However, spectroscopic techniques can only provide information about spectral data and thus limit the investigation of variabilities in wheat kernels’ appearance. To overcome this limitation, a novel approach that can combine spectral and spatial information called hyperspectral imaging technology (HSI) has attracted great attention in various research areas, especially for the classification of origins (Jin et al., 2022; Mahesh et al., 2008; Zhang et al., 2018), the detection of damages (Barbedo et al., 2015; Singh et al., 2009), and the prediction of quality attributes (Caporaso et al., 2018; Hu et al., 2021) of wheat. Caporaso et al. (2018) utilised near-infrared hyperspectral imaging (NIR HSI) within the 980–2500 nm range to predict the protein content of individual wheat grains. Similarly, Zhang et al. (2023) employed NIR HSI (969–2174 nm) to predict the protein content of wheat flour. Morales-Sillero et al. (2018) used both NIR spectroscopy (400–2498 nm) and NIR HSI (1118–2425 nm) to predict the protein content of wheat flour samples, demonstrating that the use of HSI reduced the required analysis time by at least half. In another study, Liu et al. (2022) used Fourier transform mid-infrared photoacoustic spectroscopy (FTIR-PAS) to predict protein and wet gluten content in wheat flour. Most recently, Zhang et al. (2024) integrated HSI (350–2500 nm) with machine vision to predict the protein and wet gluten content of wheat flour samples, suggesting that incorporating colour metrics could enhance prediction robustness.

Although previous studies have applied various non-destructive technologies to different datasets, there has been no direct comparison between the performance of such models as applied to spectra of grains and their corresponding flour samples. Some studies have focused exclusively on flour, while others have examined only grain, resulting in a lack of comprehensive analysis and comparability across both sample types. Consequently, a systematic evaluation of multiple spectral regions using different non-destructive technologies, applied to a unified dataset of both grain and flour samples, is essential to facilitate a direct comparison of their predictive performance. Furthermore, no prior research has specifically explored the application of HSI within the Visible (Vis) spectral range (400–700 nm) for predicting protein, wet gluten and dry gluten of wheat grains and flour. To address these gaps, this study aims to: (1) assess the performance of three non-destructive instruments—benchtop visible-near infrared hyperspectral imaging (Vis-NIR HSI), short wavelength infrared portable hyperspectral imaging (SWIR IMEC) and Fourier Transform near-infrared spectroscopy (FT-NIR MPA)—for predicting wet gluten, dry gluten and protein content in wheat kernels and their corresponding flour samples, (2) compare their predictive performance of linear and non-linear models and (3) evaluate prediction accuracy through feature selection within the Vis spectral range.

## 2 Materials and method

### 2.1 Sample preparation

A total of thirty-eight distinct wheat varieties were collected by breeders from various cities across four different geographical regions of Turkiye to ensure a broad range of variability for reference measurements. Following the removal of impurities and defective kernels, the wheat grains were stored in reusable, zipped refrigerator bags and kept under refrigeration (4 °C) until testing. For each variety, a 10 g sample of grains was randomly selected as a batch and analysed using three spectroscopic techniques. This process was repeated four times for each variety to ensure consistency. To obtain flour, each batch was ground using a household grinder (Delonghi, KG210, Treviso, Italy) and sifted through a fine mesh flour sieve (0.5 mm, The Weis, USA) for measurement using non-destructive methodologies. Upon completion of the spectroscopic analysis, the flour samples were subsequently utilised for chemical analysis.

The chemicals for gluten analysis, including potassium di-hydrogen phosphate, sodium chloride, and disodium hydrogen phosphate dihydrate were purchased from Lennox (Dublin, Ireland) and Sigma-Aldrich (Wicklow, Ireland), respectively, and stored according to their instructions.

### 2.2 Wet gluten (WG) analysis

The wet gluten contents of kernel samples were determined after milling them into the flour according to the procedure of GAFTA (GAFTA, 2003). For this purpose, 10 g of flour was placed into a porcelain mortar, and 5.5 ml of washing solution was poured drop by drop while stirring with a spatula. After forming a dough, the dough was kneaded for 5 min with a technique called rolling out and folding. The final dough was washed using the washing solution at a rate of 750 ml/8 min to remove all starch for reaching wet gluten. Subsequently, the gluten was pressed by two glass plates to eliminate excess water and weighed. The wet gluten amount is expressed as a percentage by mass of initial flour weight using the equation below:

$$\text{WG}=\frac{Wet\;gluten\;\left(g\right)\times 100}{Flour\;weight\;\left(g\right)}$$

### 2.3 Dry gluten (DG) analysis

For this purpose, wet gluten samples were put into tared Petri dishes and transferred into an oven at 105 °C for 24 h. Then, they were placed into a desiccator to cool and weighed as dry gluten (AACC, 1999).

### 2.4 Protein analysis

The protein amount of wheat samples was measured by the Kjeldahl Method which has three main steps, including digestion, neutralisation and titration, according to the AACC 46-10.01 (AACC, 1999).

### 2.5 Spectral analysis

#### 2.5.1 Visible-near infrared hyperspectral imaging system (Vis-NIR HSI)

Visible-Near Infrared (400–1000 nm with an interval of 3.19 nm) hyperspectral images of wheat kernels and their flour samples were captured by a line scanning hyperspectral camera (NEO HySpex, Oslo, Norway) in the reflectance mode. Two halogen lamps were positioned at the ∼45° to the vertical line as a lighting system, and a Spectralon white reference tile with a reflectance of 100 % was used to perform radiometric calibration.

For imaging, the power supply of the HSI system was turned on 1 h before data acquisition to equilibrate. The wheat kernels and their flours were placed on black paper with the white reference tile placed within the field of view to capture their hyperspectral images. The control of the system and image acquisition was done using HySpex Ground software (v4.9.3.8, NEO HySpex, Oslo, Norway).

#### 2.5.2 Short wavelength infrared portable hyperspectral camera system (SWIR IMEC)

For SWIR imaging, a portable IMEC SWIR Hyperspectral Snapscan camera (IMEC, Leuven, Belgium) was used with other primary components, including the spectral image sensor (InGaAs-Sensor), optics (Optec16/1.7 SWIR lens), cooling system, piezo scanning, illumination (halogen lambs), and tripod mounts. Images of wheat kernels and their flours were acquired in the wavelength range of 1100 nm–1700 nm with 99 spectral bands. Data acquisition was implemented by IMEC HSI Snapscan software (Leuven, Belgium). The lights and the system were left on for 30 min before image acquisition to avoid equipment-related noise before measurements.

#### 2.5.3 Fourier Transform near-infrared spectroscopy (FT-NIR MPA)

FT-NIR spectra of wheat and flour samples were measured using an FT-NIR spectroscopy (MPA, Bruker Optics, Ettlingen, Germany) with a 97 mm quartz cup. Diffuse reflectance spectra were recorded between 865 nm and 2540 nm (950 wavelengths) with a resolution of 16 cm^−1^ by accumulating 64 scans using an integrating sphere for both sample and background. System control and spectral acquisition were performed using the OPUS software (v. 6.5, Bruker Optics, Ettlingen, Germany).

#### 2.5.4 Spectra extraction and spectral pre-treatments

The first step of extracting spectra from hyperspectral images is removing background information. For this purpose, spectral images were displayed at each wavelength to identify the wavelength with the highest contrast between the background and the foreground. This wavelength was 787 nm for Vis-NIR HSI and 1168 nm for SWIR IMEC, respectively, and the masks were produced by thresholding the images at these wavelengths and setting background pixels to zero. The mean spectra of kernels within a given image frame were created by averaging all non-zero data related to the foreground.

Vis-NIR and SWIR spectra often show considerable differences because of physical variations between samples, including particle sizes, shape, hardness, density and heterogenous distribution of particles that can lead to light scattering effects as well as noises related to the instrument and experimental environment. To overcome these unwanted variations, different spectral pre-treatments, such as standard normal variate (SNV), Savitzky-Golay first (SG-1) and the second derivatives (SG-2) were applied in this study to compare their effect on the model performances.

#### 2.5.5 Multivariate analysis

Two different regression algorithms, partial least squares regression (PLSR) as a linear algorithm and Gaussian process regression (GPR) as a non-linear algorithm were implemented to create regression models between reference measurements and spectral data for comparing their prediction performances. PLSR is one of the most used algorithms for regression models since it is simple to implement, fast to run and has great capability to explain linear relations by transferring data into latent variables. However, the latent variable selection part is crucial, not only to eliminate model underfitting but also to overcome model overfitting. In this study, the method proposed by Gowen et al. (2011) related to the evaluation of jaggedness was used to select the optimum latent variable. The strategy behind GPR is different from PLSR because it ignores the variable space-based prediction and focuses on space-based prediction with the help of dissimilarity and affinity between samples. In other words, GPR does not try to find the best-fitting function when creating regression but tries to find a probability distribution of possible functions (Cui and Fearn, 2017).

For model development, a total of 152 mean spectra (n = 152) were utilised, with each sample consisting of 10 g of kernels or flour. Two different approaches were employed to establish the calibration and test sets. In Approach 1, the dataset was randomly partitioned, allocating 75 % of the samples (n = 114) to the calibration set and the remaining 25 % (n = 38) to the test set. This random division was performed while ensuring that each wheat variety had one representative in the test set. In Approach 2, nine wheat varieties were designated as the test set (n = 36) to validate the model's performance on previously unseen wheat varieties, while the remaining twenty-nine wheat varieties were assigned to the calibration set (n = 116). The selection of wheat varieties was informed by their reference chemical analysis to ensure a representative distribution between datasets, thereby ensuring a robust assessment of model performance. Approach 2 was employed to validate the optimal instrument and dataset identified in Approach 1. For both approaches, a 10-fold cross-validation procedure was applied to the calibration set to mitigate the risks of under-fitting or over-fitting in the models.

Bayesian Optimization with 200 iterations, utilising an acquisition function based on Expected Improvement per Second Plus, was employed to fine-tune the hyperparameters of the GPR model, specifically the basis function, kernel function and sigma. For both dataset separation approaches, the optimally tuned GPR model adopted the Matérn 5/2 kernel function, a constant basis function, and a sigma value of 8.698, without data standardisation.

Model performance was evaluated using the coefficients of the determination and the root mean square error for cross-validation (R^2^_CV_, RMSE_CV_) and for prediction (R^2^_P_, RMSE_P_), as defined in the equations below:

$$R^{2}=1-\frac{{\sum_{i=1}^{n}\;\left({\hat{y}}_{i}-y_{i}\right)}^{2}}{{\sum_{i=1}^{n}\;\left({\hat{y}}_{i}-{\bar{y}}_{i}\right)}^{2}}$$

$$\text{RMSE}=\sqrt{\frac{1}{n}{\sum_{i=1}^{n}\;\left({\hat{y}}_{i}-y_{i}\right)}^{2}}$$

where $y_{i}$ represents the actual values, ${\hat{y}}_{i}$ is the predicted values, ${\bar{y}}_{i}$ refers to the mean value of actual values and n is the sample number of the dataset. The model should have high numbers in R^2^ and low RMSE values to be considered well-performing (Özdoğan et al., 2021).

All multivariate analysis was done by MATLAB R2023b (The MathWorks, Inc., Natick, MA, USA).

#### 2.5.6 Optimal wavelength selection

Spectral images are high in dimension, with typically >100 wavelength variables. This could result in slow data processing, the need for massive data storage, and interrelatedly more investment. Therefore, selecting optimal wavelengths for multivariate analysis has gained attention to overcome these disadvantages and create models more suitable for low-cost sensor development (Liu et al., 2014).

In this study, the Neighborhood component analysis (NCA) algorithm was implemented for the selection of wavelengths after deciding the best prediction model based on the full wavelength range. It is a filter-type, non-parametric feature selection method which determines feature weights by calculating the diagonal neighborhood components to maximize the accuracy of the prediction (Kim et al., 2021).

## 3 Results and discussion

### 3.1 Sample distribution

**Fig. 1:**
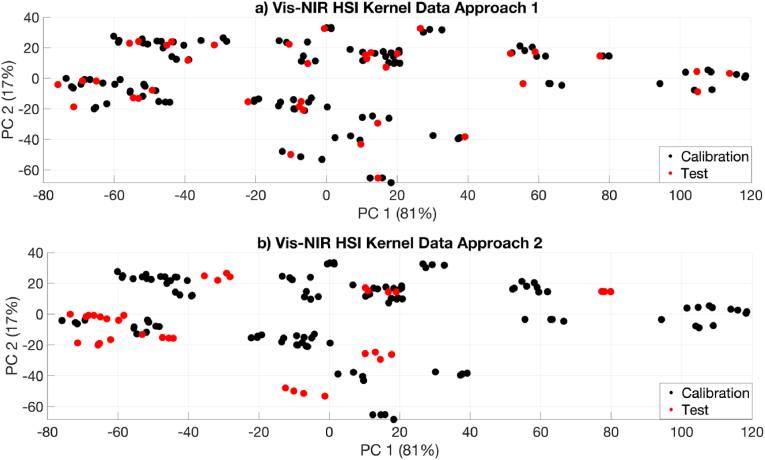
The PCA score plots of samples obtained using Vis-NIR HSI for the calibration and test set: (a) Approach 1, (b) Approach 2.

The range, mean, standard deviation (SD), coefficient of variation (CV) and statistical parameters of WG, DG and protein amount of wheat samples for calibration and test sets are shown in Table 1. A large variation in WG (CV = 30.75 %), DG (CV = 27.69 %) and protein (CV = 12.03 %) was observed in this study. This is expected because the wheat grains were from different cultivars and geographic regions, and correspondingly had different temperatures, times of rain and humidity when growing (Chen et al., 2017; Hegedűs et al., 2002). The calibration set should contain the relevant variation in all datasets to create a representative model (Liu et al., 2022). In order to assure this, both F-tests and t-tests were employed. The variance values of the calibration and test sets were assessed using F-statistics to evaluate sample variance equality before implementing a *t*-test. The results indicated no statistically significant differences between the reference measurements (i.e. WG, DG or protein) for the calibration and test datasets at a 95 % confidence level. Principal component analysis (PCA) was also carried out to inspect the variation of the spectroscopic data for the calibration and test sets as shown in Fig. 1, confirming that the calibration sets were representative. To conclude, the samples were separated appropriately to provide impartiality for model calibration.

**Table 1:** Descriptive statistics for WG, DG and protein were used to develop the calibration and prediction models.

		Subset	Range	Mean	SD	CV	F	df	P
**APPROACH 1**	**Wet Gluten**	**Calibration (n = 114)** **Test (n = 38)**	22.41–71.20 22.10–70.85	39.94 39.91	12.30 12.39	30.80 31.04	0.00	150	0.99
	**Dry** **Gluten**	**Calibration (n = 114)** **Test (n = 38)**	7.13–23.75 7.72-23.35	13.63 13.55	3.78 3.77	27.73 27.82	0.01	150	0.92
	**Protein**	**Calibration (n = 114)** **Test (n = 38)**	9.33-15.50 9.30-15.55	12.60 12.57	1.52 1.49	12.06 11.85	0.00	150	0.99
**APPROACH 2**	**Wet Gluten**	**Calibration (n = 116)** **Test (n = 36)**	22.10–71.20 26.35–58.75	40.31 38.74	13.2 8.72	32.74 22.51	2.29	89	0.41
	**Dry** **Gluten**	**Calibration (n = 116)** **Test (n = 36)**	7.13–23.75 8.81-18.88	13.70 13.31	4.01 2.91	29.27 21.86	1.89	80	0.51
	**Protein**	**Calibration (n = 116)** **Test (n = 36)**	9.33-15.50 10.09–14.91	12.58 12.69	1.59 1.28	12.61 10.10	1.53	150	0.68

Abbreviations: (n = sample number, SD = standard deviation, CV = coefficient of variation (SD/Mean∗100), df = degree of freedom of *t*-test, F= F-test value, P = p-value of *t*-test).

### 3.2 Spectral characteristics

**Fig. 2:**
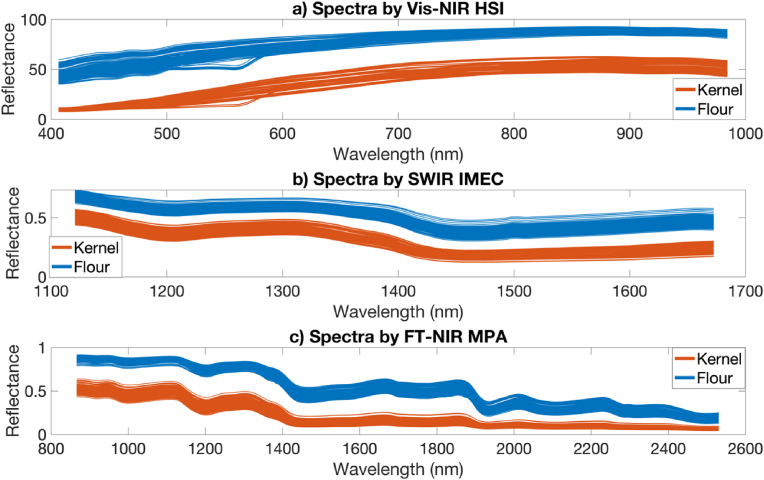
Spectra of samples obtained from different instruments.

The reflectance spectra of samples from each spectroscopic method studied are presented in Fig. 2. Although the reflectance spectra both showed the same trend for wheat kernel and wheat flour in every wavelength region, the average reflectance of the wheat kernel samples was lower than the flour samples, most likely because of the brightness of the flour compared to the kernels, due to enhanced light scattering of the smaller flour particles; this is similar to results reported in previous studies (Hu et al., 2021; Liang et al., 2020). The peak at approximately 860 nm is related to the third overtone of the C-H stretching (Liang et al., 2020). Compared to the Vis-NIR region, the SWIR region has more reflection/absorption peaks, which is expected since the SWIR region has more chemical information about the samples (Özkan et al., 2019). The peaks around 1190–1212 nm, 1446–1502 nm, 1733–1778 nm, and 1935–1952 nm are regarding the second overtone of C-H stretching vibration of samples starch and fat content, the first overtone of N-H in protein and O-H in water, the first overtone of C-H in amylose and the combination of O-H bending and stretching of water, respectively. Moreover, the peaks at regions of 2055–2060 nm and 2175–2180 nm are related to N=H and N-H bend, accordingly changes in the protein content (Tahmasbian et al., 2021).

### 3.3 Results of prediction models based on full wavelength ranges

#### 3.3.1 Approach 1: calibration and test sets spanning all wheat varieties

Both the spectra of wheat kernels and their corresponding flour spectra were used to develop regression models in Approach 1, employing PLSR and GPR for the prediction of WG, DG and protein content from wheat samples using three different spectral instruments. The results from the PLSR models are presented in Table 2, while those for the GPR models are summarised in Table 3. In general, from an algorithmic standpoint, GPR exhibited the highest coefficient of determination for prediction values (R^2^_P_) across all feature prediction models and instruments, when compared to PLSR, except for three cases: WG and protein predictions from kernel data using FT-NIR MPA and protein prediction from flour data using SWIR IMEC. The higher prediction accuracy of GPR could be attributed to its ability to capture non-linear relationships in the data through the kernel function, as well as its probabilistic framework, which enhances generalisation. The observed performance differences between these algorithms align with the findings from previous studies, such as Ong et al. (2024) on the determination of sugarcane chlorophyll content and Mayr et al. (2021) on moisture prediction in plant matrices. From an instrument-specific perspective, Vis-NIR HSI demonstrated superior performance, achieving higher coefficient of determination for prediction values (R^2^_P_) than SWIR IMEC and FT-NIR MPA for both kernel and flour datasets. These findings are consistent with our previous studies on wheat variety classification (Özdoğan and Gowen, 2025b) and grain vitreousness classification (Özdoğan and Gowen, 2025a). A possible explanation for this trend is that the visible range may contain informative spectral features relevant to predicting WG, DG and protein, which are absent in the other instruments. Furthermore, in terms of sample physical conditions, the kernel datasets exhibited better prediction performance than the flour datasets. One likely reason for that is spectral imaging can include spatial information about samples that can be useful for differentiating wheat samples, which is very efficient in this research since the kernel samples were highly varied in appearance. However, the grinding process to obtain flour samples removed the heterogeneity of the kernel samples. A similar trend was reported by Hu et al. (2021) in their study on micronutrient prediction in kernels and flour samples.

**Table 2:** Results of the PLSR algorithm utilising Approach 1.

			Vis-NIR HSI				SWIR IMEC				FT-NIR MPA			
Wet Gluten	**Kernel**	**Treatment**	**R^2^_CV_**	**RMSE_CV_**	**R^2^_P_**	**RMSE_P_**	**R^2^_CV_**	**RMSE_CV_**	**R^2^_P_**	**RMSE_P_**	**R^2^_CV_**	**RMSE_CV_**	**R^2^_P_**	**RMSE_P_**
		***Raw***	0.73	6.38	0.70	6.70	0.68	6.95	0.67	7.04	***0.70***	***6.63***	***0.82***	***5.16***
		***SNV***	0.81	5.93	0.82	5.13	***0.63***	***7.41***	***0.69***	***6.85***	0.65	7.18	0.78	5.71
		***SG-1***	***0.83***	***4.97***	***0.87***	***4.40***	0.65	7.09	0.66	7.21	0.71	6.54	0.81	5.26
		***SG-2***	0.86	4.56	0.86	4.62	0.67	7.00	0.54	8.31	0.61	7.66	0.54	8.37
	**Flour**	***Raw***	0.72	6.49	0.73	6.42	0.57	8.02	0.64	7.41	**0.75**	**6.11**	**0.75**	**6.07**
		***SNV***	0.74	6.22	0.72	6.52	0.56	8.07	0.61	7.72	0.77	5.88	0.73	6.41
		***SG-1***	***0.79***	***5.59***	***0.81***	***5.34***	**0.53**	**8.35**	**0.71**	**6.67**	0.70	6.70	0.72	6.49
		***SG-2***	0.78	5.73	0.81	5.37	0.54	8.31	0.68	6.95	0.44	9.18	0.53	8.44
Dry Gluten	**Kernel**	***Raw***	0.77	1.95	0.72	1.73	0.66	2.19	0.64	2.23	0.70	2.06	0.78	1.76
		***SNV***	***0.84***	***1.48***	***0.86***	***1.40***	0.63	2.28	0.66	2.18	0.69	2.10	0.75	1.88
		***SG-1***	0.80	1.68	0.83	1.54	***0.64***	***2.24***	***0.68***	***2.13***	***0.69***	***2.09***	***0.78***	***1.73***
		***SG-2***	0.80	1.67	0.79	1.72	0.66	2.17	0.63	2.27	0.62	2.32	0.53	2.57
	**Flour**	***Raw***	0.74	1.94	0.75	1.91	0.58	2.43	0.67	2.16	0.75	1.88	0.76	1.81
		***SNV***	0.73	1.98	0.73	1.96	0.56	2.49	0.63	2.28	***0.77***	***1.78***	***0.77***	***1.78***
		***SG-1***	0.81	1.66	0.83	1.54	***0.60***	***2.37***	***0.75***	***1.87***	0.74	1.92	0.67	2.15
		***SG-2***	***0.80***	***1.70***	***0.86***	***1.42***	0.58	2.42	0.70	2.06	0.49	2.68	0.51	2.61
Protein	**Kernel**	***Raw***	0.72	0.79	0.74	0.76	***0.74***	***0.77***	***0.85***	***0.59***	0.60	0.94	0.65	0.89
		***SNV***	0.81	0.65	0.79	0.69	0.70	0.82	0.77	0.73	0.62	0.93	0.67	0.87
		***SG-1***	0.83	0.60	0.87	0.54	0.73	0.78	0.75	0.76	0.66	0.88	0.73	0.79
		***SG-2***	***0.82***	***0.63***	***0.87***	***0.54***	0.75	0.75	0.78	0.71	***0.6***	***0.83***	***0.73***	***0.79***
	**Flour**	***Raw***	0.73	0.77	0.80	0.66	0.64	0.90	0.75	0.75	0.83	0.62	0.85	0.58
		***SNV***	0.72	0.79	0.77	0.72	0.63	0.91	0.68	0.85	***0.80***	***0.67***	***0.86***	***0.56***
		***SG-1***	0.77	0.71	0.84	0.59	***0.66***	***0.87***	***0.79***	***0.69***	0.75	0.75	0.80	0.67
		***SG-2***	***0.78***	***0.68***	***0.87***	***0.53***	0.59	0.96	0.75	0.76	0.75	0.75	0.80	0.67

(**Abbreviations:** SNV = Standard normal variate, SG-1 = Savitzky-Golay first derivative, SG-2 = Savitzky-Golay second derivative, R^2^_CV_ = Coefficient of determination for cross-validation, RMSE_cv_ = Root mean squared error for cross-validation, R^2^_P_ = Coefficient of determination for prediction, RMSE_p_ = Root mean squared error for prediction).

**Table 3:** The results of the GPR algorithm utilising Approach 1.

		Vis-NIR HSI					SWIR IMEC				FT-NIR MPA			
Wet Gluten	**Kernel**	**Treatment**	**R^2^_CV_**	**RMSE_CV_**	**R^2^_P_**	**RMSE_P_**	**R^2^_CV_**	**RMSE_CV_**	**R^2^_P_**	**RMSE_P_**	**R^2^_CV_**	**RMSE_CV_**	**R^2^_P_**	**RMSE_P_**
		***Raw***	0.92	4.15	0.94	2.96	0.81	5.35	0.85	4.64	0.35	9.90	0.43	9.30
		***SNV***	0.92	3.46	0.96	2.43	0.90	3.86	0.90	3.74	0.70	6.63	0.78	5.68
		***SG-1***	***0.92***	***2.21***	***0.97***	***1.85***	***0.91***	***3.67***	***0.95***	***2.64***	***0.76***	***5.91***	***0.79***	***5.62***
		***SG-2***	0.96	2.59	0.95	2.59	0.90	3.80	0.93	3.13	0.68	6.90	0.71	6.66
	**Flour**	***Raw***	0.34	9.95	0.43	9.34	0.35	9.90	0.37	9.80	0.57	8.02	0.74	6.26
		***SNV***	0.83	4.93	0.88	4.12	0.48	8.82	0.71	6.61	0.86	4.50	0.90	3.87
		***SG-1***	0.89	4.05	0.94	2.92	***0.64***	***7.28***	***0.80***	***5.52***	***0.86***	***4.49***	***0.92***	***3.43***
		***SG-2***	***0.90***	***3.76***	***0.94***	***2.91***	0.71	6.55	0.75	6.15	0.73	6.37	0.81	5.36
Dry Gluten	**Kernel**	***Raw***	0.90	1.15	0.95	0.79	0.77	1.80	0.84	1.47	0.36	3.01	0.42	2.86
		***SNV***	0.92	1.06	0.96	0.73	0.89	1.24	0.89	1.21	0.69	2.09	0.80	1.68
		***SG-1***	***0.96***	***0.74***	***0.98***	***0.51***	***0.90***	***1.17***	***0.94***	***0.87***	***0.75***	***1.88***	***0.80***	***1.65***
		***SG-2***	0.95	0.76	0.97	0.65	0.89	1.22	0.92	1.00	0.67	2.15	0.72	1.98
	**Flour**	***Raw***	0.83	1.54	0.92	1.02	0.37	2.98	0.41	2.89	0.54	2.55	0.73	1.92
		***SNV***	0.83	1.53	0.87	1.33	0.47	2.73	0.68	2.13	0.87	1.34	0.89	1.25
		***SG-1***	0.87	1.33	0.95	0.80	***0.65***	***2.23***	***0.76***	***1.82***	***0.87***	***1.33***	***0.93***	***0.98***
		***SG-2***	***0.90***	***1.17***	***0.95***	***0.75***	0.66	2.19	0.71	1.99	0.76	1.54	0.83	1.54
Protein	**Kernel**	***Raw***	0.90	0.47	0.93	0.40	0.79	0.68	0.81	0.65	0.21	1.34	0.25	1.32
		***SNV***	0.94	0.35	0.97	0.25	0.85	0.58	0.87	0.54	0.61	0.94	0.70	0.82
		***SG-1***	***0.97***	***0.22***	***0.98***	***0.19***	***0.89***	***0.50***	***0.94***	***0.36***	***0.67***	***0.86***	***0.77***	***0.72***
		***SG-2***	0.96	0.27	0.97	0.23	0.90	0.46	0.92	0.40	0.64	0.90	0.71	0.81
	**Flour**	***Raw***	0.17	1.37	0.19	1.37	0.20	1.35	0.22	1.34	0.61	0.94	0.61	0.95
		***SNV***	0.75	0.75	0.79	0.69	0.45	1.12	0.65	0.89	0.89	0.49	0.88	0.51
		***SG-1***	0.85	0.58	0.91	0.43	0.57	0.98	0.67	0.87	***0.90***	***0.46***	***0.88***	***0.52***
		***SG-2***	***0.88***	***0.50***	***0.97***	***0.25***	***0.55***	***1.01***	***0.75***	***0.75***	0.87	0.54	0.83	0.62

(**Abbreviations:** SNV = Standard normal variate, SG-1 = Savitzky-Golay first derivative, SG-2 = Savitzky-Golay second derivative, R^2^_CV_ = Coefficient of determination for cross-validation, RMSE_cv_ = Root mean squared error for cross-validation, R^2^_P_ = Coefficient of determination for prediction, RMSE_p_ = Root mean squared error for prediction).

For WG prediction, the highest prediction accuracy was achieved using Vis-NIR HSI, with SG-1-PLSR based on kernel data yielding an R^2^_P_ of 0.87 and RMSE_p_ of 4.40, while SG-1-GPR based on kernel data resulted in an R^2^_P_ of 0.97 and RMSE_P_ of 1.85. In a study by Ye et al. (2016), the WG of wheat was predicted using NIR spectroscopy, achieving an R^2^_P_ of 0.848 using PLSR, which is lower than the findings in the present study. This discrepancy may be attributed to the limited spectral range (850–1050 nm) employed in their study, as well as the high number of wheat varieties analysed (seventy-two), which could have introduced greater variability, thereby making generalisation more challenging. Liu et al. (2022) utilised Fourier transform mid-infrared photoacoustic spectroscopy (FTIR-PAS) to predict the WG of wheat flour samples, achieving an R^2^_P_ of 0.96 with an RMSE_P_ of 4.67 using PLSR, which is significantly higher than the results obtained in the present study using PLSR. This variation may be attributed to the differences in sample distribution, as WG content in their study ranged from 24.26 % to 29.92 %, whereas in the present study, it ranged from 22.10 % to 71.2 %. Additionally, the superior accuracy observed in their study may be due to the use of PAS, which is known to minimise light scattering and thereby enhance predictive performance, as highlighted by Hell et al. (2016). Most recently, Zhang et al. (2024) employed hyperspectral imaging in conjunction with continuous wavelet transform (CWT) and RGB imaging to extract colour features for WG prediction in flour samples. Using a Backpropagation Neural Network (BPNN), they achieved an R^2^_P_ of 0.71. However, the highest predictive accuracy (R^2^_P_ = 0.85) was obtained when combining colour features with spectral data. These findings further support the conclusion that the instrument incorporating the visible spectral region yielded higher predictive performance than others in the present study.

For DG prediction, the best prediction accuracy was achieved using Vis-NIR HSI combined with SNV-PLSR on kernel data, resulting in an R^2^_P_ of 0.86 and RMSE_p_ of 1.40, whereas SG-1-GPR on kernel data yielded an R^2^_P_ of 0.98 and RMSE_P_ of 0.51. In a study of Başlar and Ertugay (2011), NIR spectroscopy was employed to predict the DG content of wheat flour samples, achieving a prediction accuracy of 0.953 using Multiple Linear Regression (MLR). The accuracy differences between studies may be attributed to the conditioning of moisture in the samples prior to analysis, a step not included in our study, as well as differences in the algorithms used. Similarly, Miralbés (2003) reported an R^2^_P_ of 0.97 for dry gluten prediction using modified PLSR, utilising NIR transmittance spectroscopy on seventeen wheat varieties. This superior predictive accuracy in that study may be due to the transmittance mode, which allows light to pass through the sample and interact with a greater volume of the material, thereby reducing spectral scattering and multiple reflections, ultimately leading to lower noise, as demonstrated in the study of Wang et al. (2005) for the comparison of reflectance and transmission modes for determining wheat kernel vitreousness.

For protein prediction, both kernel and flour data yielded the same prediction accuracy, with an R^2^_P_ of 0.87 when using SG-2 combined with PLSR by Vis-NIR HSI. However, greater accuracy was observed when GPR was applied: kernel data pre-processed with SG-1 achieved an R^2^_P_ of 0.98, while flour data reached an R^2^_P_ of 0.97. In comparison, Caporaso et al. (2018) employed SWIR-HSI to predict the protein content in individual kernels, achieving a lower R^2^_P_ of 0.79 using PLSR. The reduced performance could be attributed to differences in sample type—individual kernels versus bulk samples in the current study—and the exclusion of the visible spectral region in their analysis. Similarly, Ye et al. (2018) utilised the NIR grain quality analyser, The InfraTec 1241, and attained a remarkably high R^2^_P_ of 0.99 with PLSR. This superior performance may result from their use of transmission mode, which generally provides more penetrating spectral data, rather than the reflectance mode used in the present study. Shuqin et al. (2016) used NIR (900–1700 nm) HSI to predict protein content of wheat grains, using both PLSR and Radial Basis Function Neural Network (RBF-NN). Their models achieved R^2^_P_ values of 0.85 and 0.92, respectively. These results reinforce the observed performance gap between linear and non-linear algorithms in our study, suggesting that spectral data may exhibit non-linear relationships with protein content, which non-linear models like GPR and RBF-NN can better capture.

Spectral pre-treatments generally had a positive impact on the predictive performance of the models. In particular, SG derivatives achieved the highest accuracy, suggesting that the dataset contains a certain degree of noise and fluctuations that are challenging to interpret without pre-processing. However, in three specific—protein prediction from kernel data using SWIR IMEC, and WG prediction from kernel and flour data using FT-NIR MPA—the raw spectral data outperformed the pre-treated versions. This observation may indicate that, for these datasets, the pre-processing steps inadvertently removed relevant information during the denoising process (Amigo et al., 2013). When GPR employed, spectral pre-treatment had a notably positive effect on flour data derived from spectral imaging techniques, whereas a similar improvement was observed in kernel data when FT-NIR MPA was used. This disparity might be attributed to the physical characteristics of wheat kernels; FT-NIR spectroscopy typically collects data from a single point, which can increase scattering and thus affect model performance (Caporaso et al., 2018). Conversely, converting samples to flour may enhance spectral reflectance due to increased brightness, especially when hyperspectral imaging is employed (Hu et al., 2021).

Overall, the highest predictive accuracies for the prediction of WG, DG and protein content were achieved using kernel datasets combined with SG-1, obtained through Vis-NIR HSI. Accordingly, Approach 2 was adopted for external validation, employing these datasets to predict wheat varieties that were not included in the model development phase. This approach also facilitated a comparison of the predictive performance for WG, DG and protein content prediction between linear and non-linear models when applied to previously unseen wheat varieties.

#### 3.3.2 Approach 2: calibration and test sets spanning different wheat varieties

The prediction results obtained using PLSR and GPR through Approach 2, for both raw and SG-1 pre-treated data, are summarised in Table 4. For raw kernel data, both PLSR and GPR models yielded R^2^_P_ values below 0.57 for the prediction of WG, DG and protein content—except for the GPR model predicting protein content, which achieved an R^2^_P_ of 0.76. This highlights the significance of selecting an appropriate algorithm to accurately capture the underlying relationships within the datasets. The GPR model based on raw data using Approach 1 achieved a higher R^2^_P_ of 0.93. This was anticipated, as the model was trained using data from all varieties, thereby improving generalisation within known varieties and resulting in increased prediction accuracy. Nevertheless, the results obtained from Approach 2 offer a more realistic estimate of the models’ performance in practical scenarios, particularly when encountering previously unseen varieties. Therefore, the choice of selection of data partitioning strategy is crucial consideration in developing robust models that are suitable for their intended application.

**Table 4:** The results of prediction models utilising Approach 2.

		PLSR				GPR			
	Treatment	R^2^_CV_	RMSE_CV_	R^2^_P_	RMSE_P_	R^2^_CV_	RMSE_CV_	R^2^_P_	RMSE_P_
**WET GLUTEN**	*Raw*	0.70	7.24	0.20	7.83	0.90	4.22	0.45	6.44
	***SG-1***	**0.85**	**5.04**	**0.56**	**5.78**	**0.97**	**2.22**	**0.83**	**3.60**
**DRY GLUTEN**	*Raw*	0.73	2.08	0.27	2.49	0.94	1.02	0.57	1.91
	***SG-1***	**0.83**	**1.65**	**0.73**	**1.51**	**0.97**	**0.68**	**0.77**	**1.39**
**PROTEIN**	***Raw***	**0.81**	**0.66**	**0.12**	**1.20**	**0.95**	**0.36**	**0.76**	**0.63**
	*SG-1*	0.87	0.57	0.65	0.76	0.99	0.17	0.69	0.71

(**Abbreviations:** SG-1 = Savitzky-Golay first derivative, R^2^_CV_ = Coefficient of determination for cross-validation, RMSE_cv_ = Root mean squared error for cross-validation, R^2^_P_ = Coefficient of determination for prediction, RMSE_p_ = Root mean squared error for prediction, PLSR = Partial Least Square Regression, GPR = Gaussian Process Regression).

Using SG-1 pre-treated data in conjunction with GPR, the coefficient of determination for the prediction of WG and DG contents increased drastically, to 0.83 and 0.77, respectively. These results underscore the significance of selecting appropriate spectral pre-treatment methods to enhance the performance of multivariate predictive models. This finding is consistent with the study conducted by Mishra and Lohumi (2021), whom explored various spectral pre-treatment techniques to improve the predictive accuracy of models for protein content of wheat grains. However, the application of SG-1 pre-treatment resulted in a slightly reduced R^2^_P_ value (from 0.76 to 0.69) for protein prediction using GPR, suggesting that some critical information related to protein content may have been lost during the pre-treatment process.

Across all features, for both raw and SG-1 treated datasets, GPR consistently outperformed PLSR in terms of R^2^_P_. This indicates a non-linear relationship between the spectral data and compositional parameters in wheat grains. Nevertheless, the performance gap between PLSR and GPR was smaller for DG and protein predictions using SG-1-treated data, implying that the relationship between WG and the spectral data is potentially more non-linear in nature compared to DG and protein content.

Overall, Approach 1 demonstrated superior predictive accuracy compared to Approach 2, as anticipated. Although the test sample differed entirely from the calibration set, the presence of all varieties within the calibration set meant that the test data were not entirely novel in the context of Approach 1. In contrast, Approach 2 involved a more stringent evaluation, wherein the test set included varieties that were not present in the calibration phase. Nonetheless, the results from Approach 2 indicate that the predictive models relied on generalized features related to WG, DG, and protein content, rather than on variety-specific patterns. The decline in model performance observed in Approach 2 highlights the significance of incorporating a broader diversity of wheat varieties. Increasing the variability within the dataset is essential for enhancing the robustness and predictive accuracy of the developed models.

### 3.4 Results of prediction models based on selected wavelengths in the visible range

**Fig. 3:**
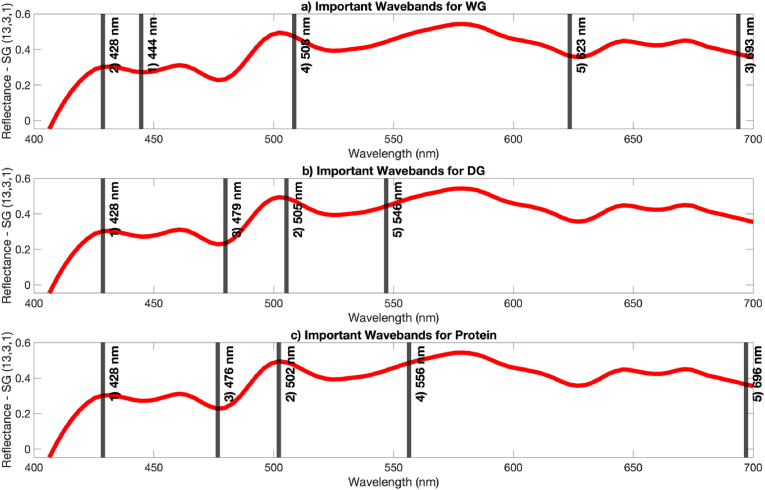
Selected wavebands within the Visible region (SG-1 treated) according to their importance for wheat grain datasets.

The optimal results were achieved by GPR using Vis-NIR HSI. Consequently, optimal wavelength selection using NCA was applied to these datasets. In order to explore this further, the Vis-NIR HSI wavelength range was narrowed to the visible spectrum, specifically from 400 to 700 nm, for wavelength selection. The wavelengths selected according to their importance, based on SG-1 treated kernel datasets, are presented in Fig. 3, with the regression model performances using GPR shown in Table 5.

**Table 5:** Results of the GPR based on selected wavelengths.

		Sample	Treatment	Selected WL	R^2^_CV_	RMSE_CV_	R^2^_P_	RMSE_P_
APPROACH 1	**Wet Gluten**	Kernel	Raw	489, 700, 412, 527	0.88	4.24	0.95	2.77
		Kernel	SG-1	444, 428, 693, 508	0.94	2.93	0.97	2.16
		Flour	SG-1	425, 502, 483, 505	0.83	5.15	0.91	3.74
	**Dry Gluten**	Kernel	Raw	700, 530, 489, 412	0.89	1.24	0.95	0.84
		Kernel	SG-1	428, 505, 479, 700	0.95	0.87	0.97	0.69
		Flour	SG-1	425, 502, 483, 447	0.84	1.52	0.95	0.84
	**Protein**	Kernel	Raw	412, 486, 530, 700	0.90	0.49	0.93	0.42
		Kernel	SG-1	428, 502, 476, 556	0.96	0.32	0.98	0.19
		Flour	SG-1	425, 498, 447, 483	0.85	0.61	0.87	0.57
APPROACH 2	**Wet Gluten**	Kernel	SG-1	444, 428, 693, 508, 623	0.96	2.51	0.71	4.74
	**Dry Gluten**	Kernel	SG-1	428, 505, 479, 700, 546	0.96	0.76	0.71	1.56
	**Protein**	Kernel	SG-1	428, 502, 476, 556, 696	0.98	0.22	0.82	0.55

(**Abbreviations:** SG-1 = Savitzky-Golay first derivative, R^2^_CV_ = Coefficient of determination for cross-validation, RMSE_cv_ = Root mean squared error for cross-validation, R^2^_P_ = Coefficient of determination for prediction, RMSE_p_ = Root mean squared error for prediction).

In Approach 1, it is evident that the selected wavelengths from the kernel and the flour data were largely similar, with only slight differences. The important wavelengths identified were around 425, 445, 480, 500, 555, and 700 nm, which correspond to violet, blue, blue, green, yellow and red, respectively (Helmenstine, 2023). These wavelengths are all within visible range, suggesting a potential relationship between the visible spectra and WG, DG, and protein content in wheat grains. The wavebands at 498, 470 and 648 nm were previously associated with chlorophyll and carotenoid content in wheat, as reported by Hernandez et al. (2015), which corroborates earlier studies suggesting a correlation between pigment ratios and protein content (Hailu and Merker, 2008; Jin et al., 2014). Predictions based on raw flour data were excluded from the results, as the performance of the algorithm was deemed unacceptable following the selection of only four wavelengths. The best predictive models were derived from kernel data with SG-1 treatment, which mirrored the models based on the full wavelength range. Furthermore, the models did not experience a reduction in performance when using only four wavelengths compared to the full wavelength models, and in fact, demonstrated more accurate cross-validation results. This suggests that the selected wavelengths provided relevant information while eliminating collinear and redundant data, thus mitigating model overfitting (Zhang et al., 2020). The highest prediction performances based on kernel data with four selected wavelengths using GPR were R^2^_P_ of 0.97 and RMSE_P_ of 2.16 for WG, R^2^_P_ of 0.97 and RMSE_P_ of 0.69 for DG and R^2^_P_ of 0.98 and RMSE_P_ of 0.19 for protein. These results surpass those reported in previous studies (Caporaso et al., 2018; Shuqin et al., 2016) and align closely with the research conducted by Albanell et al. (2012).

In Approach 2, the models using four selected wavelengths produced R^2^_P_ values below 0.70. As a result, the number of selected wavelengths was increased to five. The coefficient of determination (R^2^_P_) for WG and DG predictions decreased in comparison to the models based on the full wavelength range; however, remained above an acceptable level (R^2^_P_ > 0.70). Remarkably, the R^2^_P_ value for protein prediction improved from 0.69 to 0.82 with wavelength selection. This indicates the significance of selecting the most informative features to develop more robust regression models. These findings suggest that WG, DG, and protein content in wheat grains can be predicted with an accuracy exceeding R^2^_P_ of 0.70 using five wavelengths within the visible range, even when applied to previously unknown wheat varieties. This underscores the relevance of the selected spectral features and the significance of the visible range for compositional analysis in the current study.

Overall, the findings present several key implications: (1) Using visible light is more cost-effective than utilising other spectral regions, (2) The visible range could facilitate the development of portable devices to assess wheat grain quality in the field or during intake, aiding real-time decision-making, and (3) The visible range enables the use of low-cost, compact sensors (such as LED-based systems) for widespread deployment.

## 4 Conclusions

A dataset (152 samples) from 38 different wheat cultivars and their corresponding whole-grain flour samples were presented in this study, and the research has represented the feasibility of non-invasive prediction of their WG, DG and protein contents using spectral imaging (Vis-NIR HSI and SWIR IMEC) and spectroscopic (FT-NIR MPA) techniques. Through comparison, the Vis-NIR HSI was found as the highest-performance equipment for the WG, DG and protein predictions. Moreover, the spectral data obtained from wheat kernels produced more accurate predictions than those derived from flour samples. Non-linear models, particularly GPR, outperformed linear models such as PLSR in terms of predictive accuracy. Beyond models developed using the full wavelength range, wavelength selection via NCA was performed within only the visible range (400–700 nm). Remarkably, models utilising only four selected wavelengths achieved R^2^_P_ values above 0.97 for all reference measurements. These findings suggest a significant underlying relationship between the visible spectral characteristics of wheat samples and their protein and gluten content. Nonetheless, the study has certain limitations: (1) the samples originate from a single country, limiting geographical variability, (2) the imaging equipment employed is benchtop and designated for laboratory use, (3) the number of wheat varieties is relatively small compared to the global diversity of wheat grains. To address these constraints, future studies should aim to include a broader range of wheat varieties from diverse geographical regions and assess the applicability and performance of portable HSI devices for practical, real-world deployment.

## Author statements

•it is our original work that does not contain plagiarism as a whole or in parts and is not currently being submitted/processed in other journals.
•we have obtained written permission for unrestricted use of any previously copyrighted material included in the article.
•all authors have made significant contribution to the work and have agreed with the final version to be published.
•the study was conducted in full compliance with the journal Current Research in Food Science's ethics guidelines.
•we take full responsibility for the accuracy of the data and the report of the work should problems occur in the future.

## Declaration of competing interest

The authors declare the following financial interests/personal relationships which may be considered as potential competing interests:

Gozde Ozdogan reports financial support was provided by Republic of Turkey Ministry of National Education. Gozde Ozdogan reports a relationship with Republic of Turkey Ministry of National Education that includes: funding grants. If there are other authors, they declare that they have no known competing financial interests or personal relationships that could have appeared to influence the work reported in this paper.

## Data Availability

The data that has been used is confidential.
